# Supplementary material for: Multi-wavelength spin dynamics of defects in hexagonal boron nitride
Source: Light Sci Appl. 2026 Jun 25;15:283. doi: 10.1038/s41377-026-02398-z (PMC13303940; doi:10.1038/s41377-026-02398-z)
Supplement: Supplementary file 1 — Supporting Information for Multi-wavelength Spin Dynamics of Defects in Hexagonal Boron Nitride [file 41377_2026_2398_MOESM1_ESM.pdf]

Supporting Information for Multi-wavelength Spin Dynamics of Defects in Hexagonal Boron Nitride

Ivan Zhigulin[†,1,2], Nicholas P. Sloane[†,*,1,2], Benjamin Whitefield[1,2], Konosuke Shimazaki[1,2], Jean-Philippe Tetienne[3], Mehran Kianinia[*,1,2], and Igor Aharonovich[1,2,4]

[1] School of Mathematical and Physical Sciences, University of Technology Sydney, Ultimo, New South Wales 2007, Australia
[2] ARC Centre of Excellence for Transformative Meta-Optical Systems, University of Technology Sydney, Ultimo, New South Wales 2007, Australia
[3] Department of Physics, School of Science, RMIT University, Melbourne, VIC 3001, Australia
[4] Department of Physics, Kyung Hee University, Seoul 02447, Republic of Korea

† These authors contributed equally to this work.
* To whom correspondence should be addressed: N.P.S. Nicholas.sloane@uts.edu.au, M.K. Mehran.Kianinia@uts.edu.au

Section 1: Spin Hamiltonian of the spin complex.

The total spin Hamiltonian (H_{tot}) of the spin complex emitter can be described as the sum of the individual Hamiltonians of the strongly coupled spin pair (H_S) and the weakly coupled spin pair (H_W):

$$H_{tot} = H_S + H_W$$
$$H_S = D(\hat{S}_z{}^2 - \frac{S}{3}(S+1)) + E(\hat{S}_x{}^2 + \hat{S}_y{}^2) + g_e \mu_B \vec{B} \cdot \vec{S}$$
$$H_W = g_e \mu_B \vec{B} \cdot \vec{S}$$

Where D and E are the axial and transverse zero-field splitting (ZFS) parameters, respectively, $g_e = 2$ is the electron g-factor, μ_B is the Bohr magneton, $\vec{B}$ is the applied magnetic field vector, and $\vec{S} = (\hat{S}_x, \hat{S}_y, \hat{S}_z)$ is the vector of spin-1 operators. While the Hamiltonian of the weakly coupled spin pair is influenced only by the Zeeman interaction, the strongly coupled spin pair is affected by both the Zeeman interaction and the ZFS arising from stronger dipolar interactions between the individual spin-½ particles. The eigenvalues of the Hamiltonians of the strongly and weakly coupled spin pairs are shown in Figure SI1a,b, respectively. Additionally, the simulated optically detected magnetic resonance (ODMR) transitions at different magnetic fields, determined from these eigenvalues, are shown in Figure SI1c.

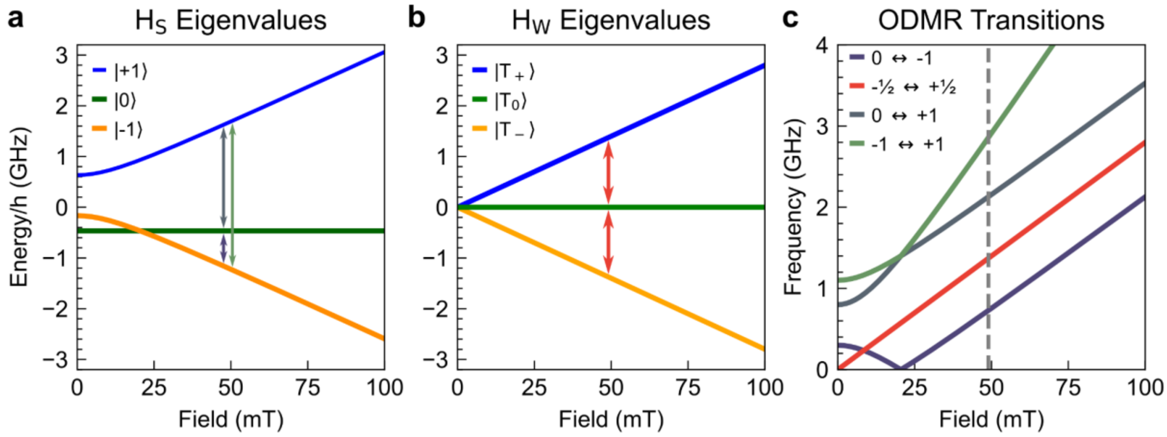

Figure SI1: Calculated eigenvalues and ODMR transitions from spin Hamiltonian. *Calculated eigenvalues at different external magnetic field strength for the Hamiltonians **(a)** H_S and **(b)** H_W. **(c)** Simulated ODMR transitions at different magnetic fields based on the transitions between the different eigenvalues shown in **(a)** and **(b)**.*

Section 2: Additional data of spin complex emitter with 721 nm ZPL.

Figure SI2a shows a confocal raster scan of a hBN flake. Dashed circle indicates position of the emitter studied in the manuscript. Majority of other bright spots correspond to optically active hBN emitters. Figure SI2b shows photoluminescence (PL) of the emitter excited either with 532 nm or with 633 nm, with both spectra showing no discernible difference between each other.

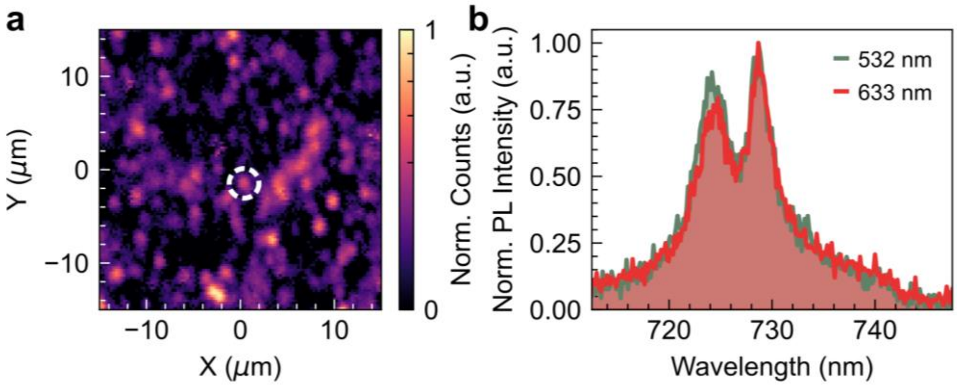

Figure SI2: Confocal scan and spectra of emitter with 721 nm ZPL. (a) *Confocal scan highlighting the location of the emitter (dashed circle).* ***(b)*** *PL spectra of the emitter under 532 nm (green) and 633 nm (red) excitation.*

To quantify the type of emission we conducted second order autocorrelation measurements that involved splitting PL photons with 50:50 probability into two separate avalanche photodiode detectors. A time correlator card was used to build a bi-directional histogram that revealed strong antibunching at small delay times ($\tau < 2$ ns), signifying sub-poissonian statistics of photon emission. We conducted this analysis for the same emitter at half the saturation power for 532 nm and 633 nm excitation wavelengths with both falling below 0.5 (0.39 and 0.42), satisfying single emitter criterion. Besides the small changes in the $g^{(2)}(0)$ values for each excitation wavelength, photon statistics, importantly, reveal larger bunching amplitudes of the shelving state. This was discussed in the main text of this work and also accounts for the observed blinking under 633 nm excitation from this emitter.

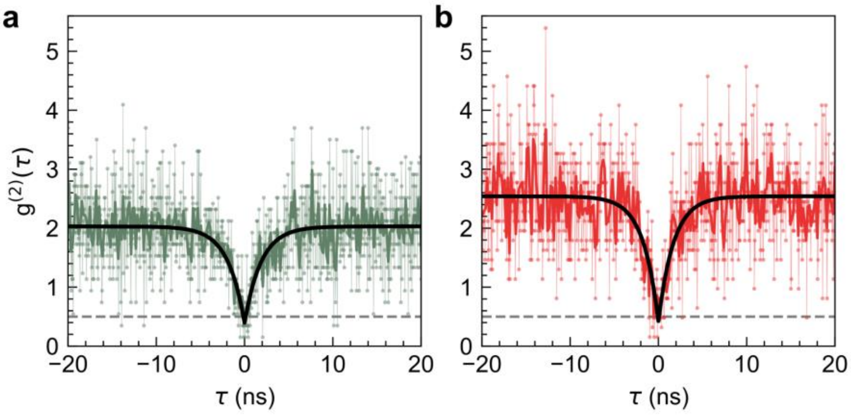

Figure SI3: Autocorrelation measurements of emitter with 721 nm ZPL. *Autocorrelation measurements of the emitter shown in Figure SI2 under* ***(a)*** *532 nm and* ***(b)*** *633 nm excitation. The extracted $g^{(2)}(0)$ values are 0.39 and 0.42 for 532 nm and 633 nm excitation, respectively.*

In Figure 2a and b of the main text we show continuous-wave (CW)-ODMR spectra and the power dependent ODMR contrasts of the -½ ↔ +½ and 0 ↔ +1 transitions, each corresponding to either S = {1,0} or S = 1 spin manifolds. However, since the S = 1 also exhibits 0 ↔ -1 and -1 ↔ +1 transitions, we performed similar analysis for those here. Results are summarised in Figure SI4. Similarly, a change of excitation wavelength has a drastic effect on the ODMR contrast of both transitions, with 633 nm tripling the intensity of -1 ↔ +1. Furthermore, Figure SI4b shows that the transitions exhibit the same trend as a function of optical excitation power, following an initial increase and subsequent decrease in contrast at high powers.

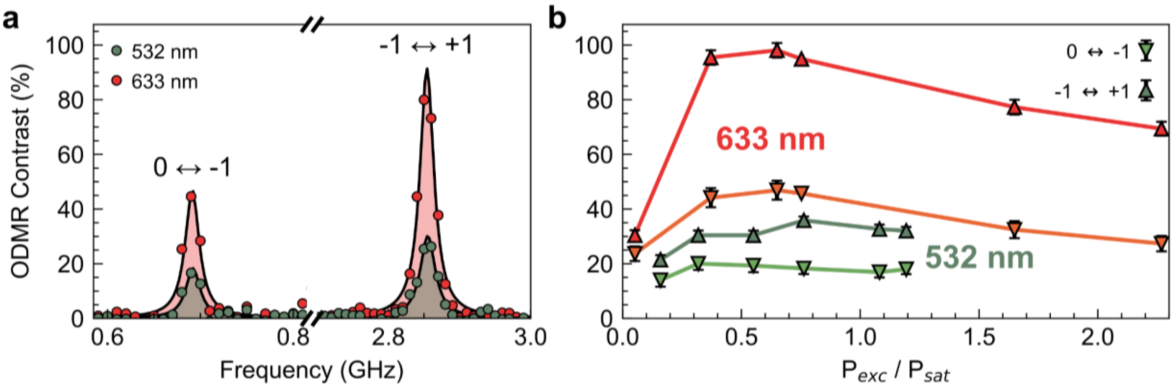

Figure SI4: Other ODMR transitions of S = 1 of emitter with 721 nm ZPL. (a) *CW-ODMR spectra showing relative contrast of the 0 ↔ -1 and -1 ↔ +1 transitions under 532 nm and 633 nm excitations, corresponding to the emitter presented in main text and in Figure SI2.* ***(b)*** *Measured ODMR contrast of the 0 ↔ -1 (∇) and -1 ↔ +1 (Δ) transitions under 532 nm and 633 nm excitation plotted as a function of the excitation power normalised by the saturation power (P_{exc}/P_{sat}).*

To normalise optical excitation powers for each excitation wavelength, ODMR contrasts in Figure 2b and in Figure SI4 were plotted as a function of normalised power. This first required extracting saturation powers P_{sat} for 532 nm and for 633 nm using corresponding datasets.

These saturation curves are presented in Figure SI5 follow the standard power saturation model described in the main text, except without the constant offset. The updated model (written in caption of Figure SI5) yields saturation powers of 151 μW and 97 μW for 532 nm and 633 nm excitations, respectively.

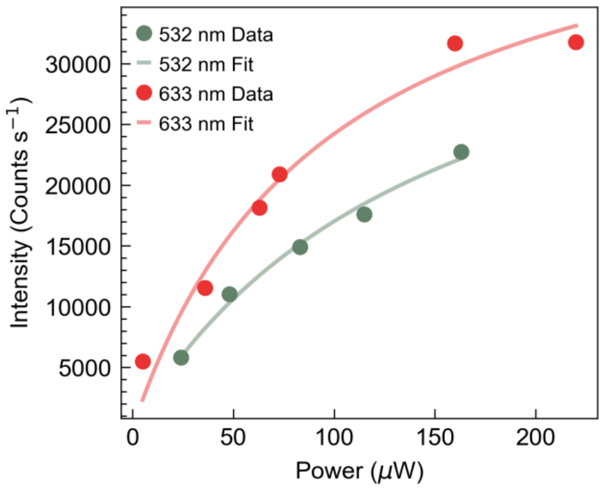

Figure SI5: Power saturation of the emitter with 721 nm ZPL. *Optical power saturation data under 532 nm and 633 nm excitations of the emitter shown in Figure SI2. Solid lines correspond to fits using the model* $I(P) = I_\infty \cdot P/(P + P_{sat})$. *The extracted saturation powers are 151 μW and 97 μW for 532 nm and 633 nm excitations, respectively.*

To further investigate the different photodynamics exhibited by the emitter under 532 nm and 633 nm excitation, long-timescale second-order autocorrelation measurements were performed (Figure 2e), which are well fit by a four-level system of the form:

$$g^{(2)}(\tau) = A(1 - (\wp + B)e^{-t/\tau_{rad}} + \sum_{i=1}^{3} B_i\, e^{-t/\tau_i})$$

Where A is the amplitude, $\wp$ is the purity, B is the total bunching amplitude ($B_1 + B_2 + B_3$), τ_{rad} is the excited-state lifetime, B_i are the individual bunching amplitudes, and τ_{i} are the individual bunching lifetimes.

The relative lifetimes and amplitudes extracted from fitting the long-timescale second-order autocorrelation measurements for both 532 nm and 633 nm excitation are summarised in Table SI1. The excited-state lifetimes are similar for both excitation wavelengths. However, as shown in Figure 2e, the relative bunching amplitudes associated with the intermediate states are significantly larger under 633 nm excitation compared to 532 nm, indicating stronger coupling from the excited state accessed at 633 nm into metastable or shelving states. In particular, B_1 increases significantly from 0.4 (532 nm) to 3.6 (633 nm), suggesting substantially more coupling from the excited states accessed by 633 nm excitation to longer lived metastable states.

Furthermore, both excitation wavelengths populate long-lived states, with lifetimes of 64 μs (532 nm) and 463 μs (633 nm). These long timescales are consistent with charge-state switching dynamics. The substantially longer lifetime observed under 633 nm excitation

suggests that the associated charge states are more stable, which may explain the blinking behaviour observed in the time traces of the emitter PL (Figure 2d).

Table SI1: Extracted lifetimes from long second order correlation measurement of the emitter with 721 nm ZPL.

Excitation Wavelength	τ_{rad} (ns)	B_1	τ_1 (ns)	B_2	τ_2 (ns)	B_3	τ_3 (μs)
532 nm	1.8	0.4	576	0.5	3529	0.03	64
633 nm	1.7	3.6	163	1.1	1490	0.025	463

Next, we examine the behaviour of the emitter with a 721 nm ZPL under pulsed ODMR measurements. Rabi oscillations are first measured using the pulse sequence shown in Figure SI6a. Consistent with the continuous-wave measurements, we observe an increased ODMR contrast under 633 nm excitation compared to 532 nm excitation. From these rabi measurements the π-pulse durations for both the −½ ↔ +½ and 0 ↔ +1 spin transitions can be determined. Following this, readout-delay measurements were performed using the pulse sequence shown in Figure SI6b, in which the initialization and readout laser pulses are separated by a π-pulse and the delay (τ) between the reference and signal readout windows is swept. The results of this measurement, for both excitation wavelengths and for the −½ ↔ +½ and 0 ↔ +1 transitions, are shown in Figure SI6e–h. In all cases, two main features are observed: (i) an initial spike followed by a decay in both the reference and signal traces, and (ii) a component in the signal trace that grows in amplitude before decreasing at longer delay times. The first component is attributed to population initially in the ground state, giving rise to a strong PL signal that decays as population is transferred to metastable/shelving states[1]. The second component arises from the π-pulse transferring population into a shorter-lived state prior to readout, which subsequently repopulates the ground state and leads to an increase in fluorescence. The contrast eventually decreases to zero as the system is reinitialized by the readout pulse. This results in an increased calculated contrast, consistent with the CW-ODMR measurements. Interestingly, the time required for the contrast to reach its maximum is longer under 633 nm excitation than under 532 nm excitation (approximately 450 ns and 350 ns for 633 nm and 532 nm excitation, respectively, for the −½ ↔ +½ transition). These results raise further questions regarding spin initialization and polarisation following intersystem crossing, and the extent to which these processes depend on the different excited states accessed under different excitation wavelengths.

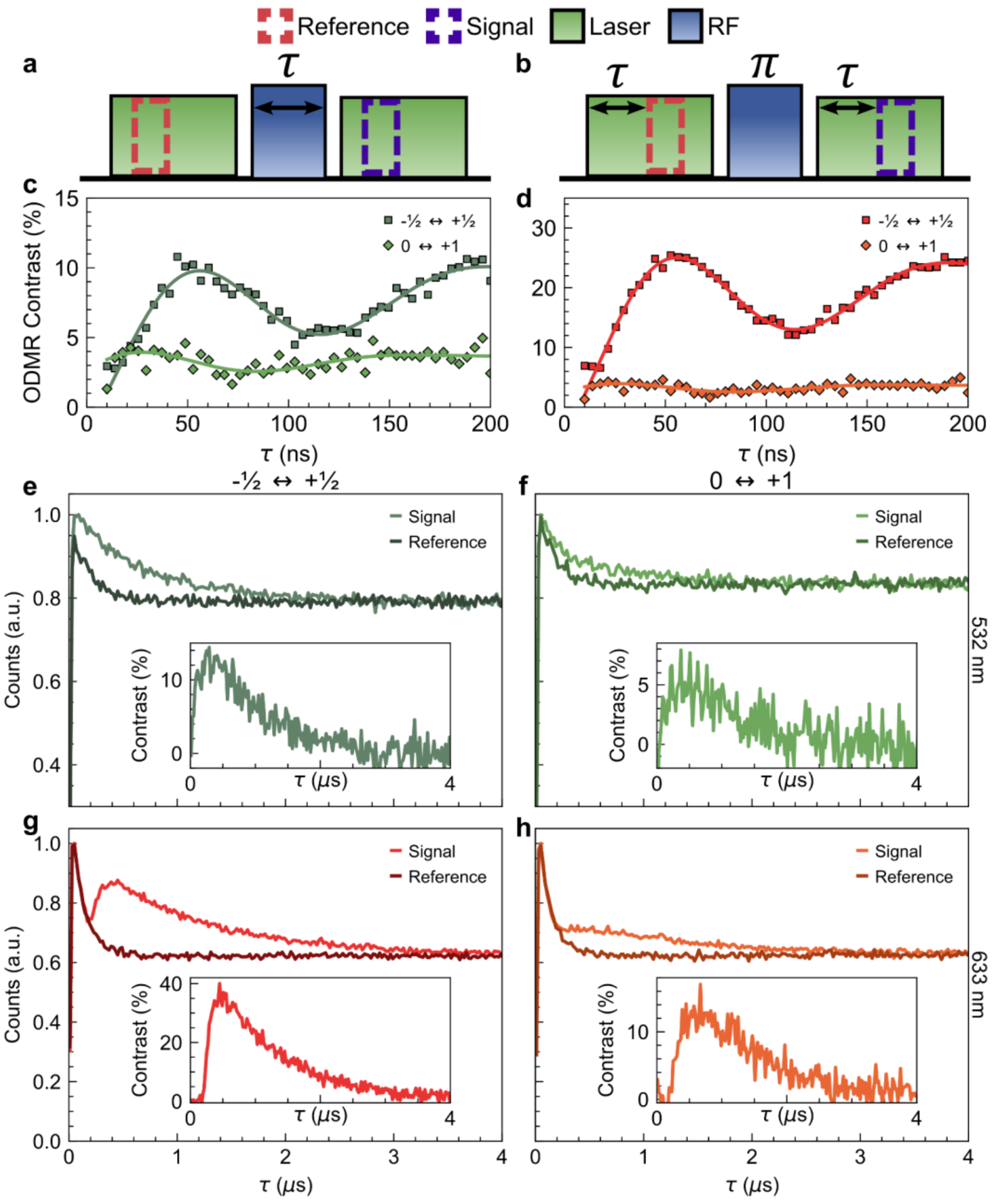

Figure SI6: Rabi oscillations and readout delay of emitter with 721 nm ZPL. *(a) Pulse sequence for Rabi oscillation measurements, consisting of an initialization laser pulse, followed by an RF pulse of variable duration (τ), and a final readout laser pulse. (b) Pulse sequence for readout-delay measurements, consisting of an initialization and readout laser pulse separated by a π-pulse. The readout gate offset is varied in time (τ), as indicated. Dashed rectangles denote gated signal integration windows for the reference (red) and signal (purple) channels used to extract the ODMR contrast. (c–d) Rabi oscillations of the emitter under 532 nm (c) and 633 nm (d) excitation for the −½ ↔ +½ and 0 ↔ +1 spin transitions. (e–f) Readout-delay measurements under 532 nm excitation for the −½ ↔ +½ (e) and 0 ↔ +1 (f) transitions. (g–h) Readout-delay measurements under 633 nm excitation for the −½ ↔ +½ (g) and 0 ↔ +1 (h) transitions. Insets in (e–h) show the corresponding ODMR contrast as a function of readout delay time (τ).*

Section 3: Excitation wavelength-dependent behaviour of other spin complex emitters.

In addition to the emitter discussed in the main text and in Section 2 above, other spin complex emitters in hBN were also investigated. We begin with an emitter that has ZPL at 703 nm. Both the PL spectra and autocorrelation measurements of this emitter are shown in Figure SI7a-c for 405 nm, 532 nm, and 580 nm excitations. We note that this emitter could not be excited above 600 nm. Also shown in Figure SI7d is the CW-ODMR spectra of the three different used excitation wavelengths. No detectable ODMR signal was observed under 405 nm excitation, whereas both 532 nm and 580 nm both showed transitions from S = 1 and S = {1,0} manifolds. The absence of a detectable ODMR signal under 405 nm excitation may be attributed to charge state switching or photoionization, resulting in a spin-inactive charge configuration. Alternatively, as observed in the spectrum in Figure SI7a 405 nm excitation appears to also excite other emissive species, potentially reducing the ODMR contrast of the emitter of interest below the noise floor. From those results, we observe far stronger ODMR contrast of the emitter when excited with 532 nm, compared to the measured signal under 580 nm excitation. Because of that, power dependence of only the 532 nm excitation could be meaningfully measured, since the signal-to-noise ratio of the 580 nm spin transitions is very low for reliable analysis. We find that, similar to the emitter investigated in the main text, the contrast depends on optical excitation power (Figure SI7e), where the ODMR signal exhibits initial increase followed by a steady decrease at higher powers. This behaviour is consistent with the conceptual model proposed in Figure 2f of the manuscript and is discussed further in Supplementary Section 4.

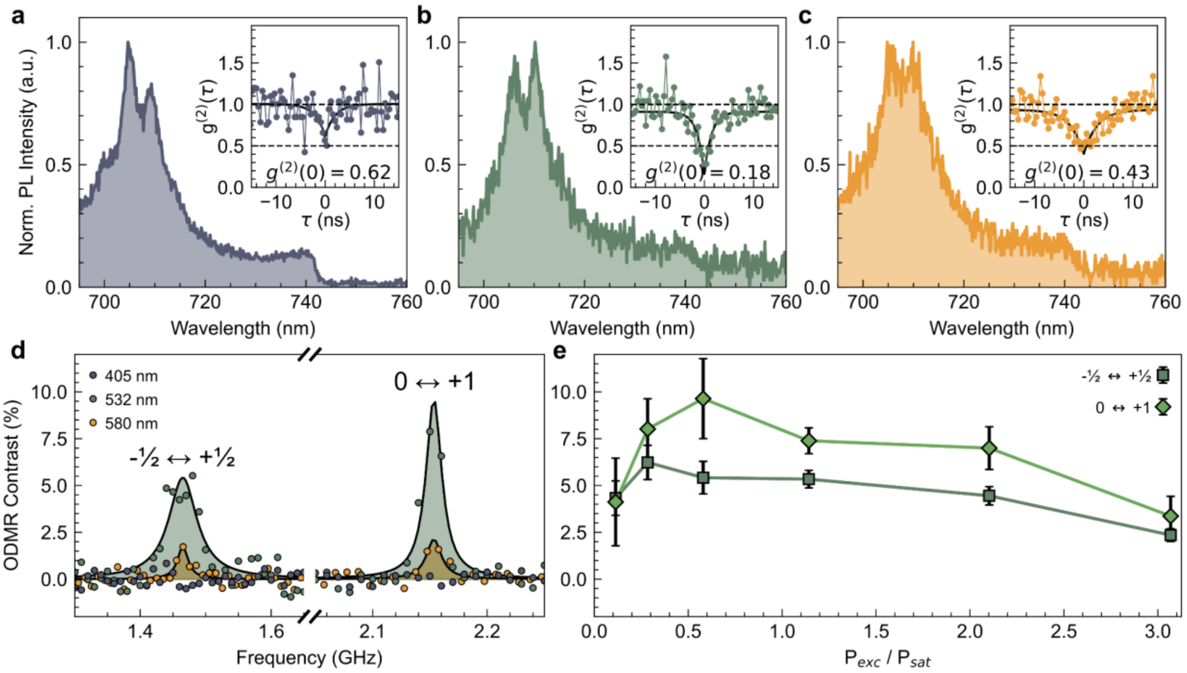

Figure SI7: PL and ODMR of emitter with 703 nm ZPL. *PL spectra of the emitter under* ***(a)*** *405 nm (blue),* ***(b)*** *532 nm (green), and* ***(c)*** *580 nm (orange) excitations. Inset: autocorrelation measurements under the corresponding wavelength excitations.* ***(d)*** *CW-ODMR spectra of the emitter excited with 405 nm, 532 nm, and 580 nm showing the relative contrast for the -½ ↔*

+½ and 0 ↔ +1 transitions. For the 405 nm excitation no ODMR was detected. ***(d)*** *Measured ODMR contrast of the -½ ↔ +½ (squares) and 0 ↔ +1 (diamonds) transitions under 532 nm excitation plotted as a function of the excitation power normalised by the saturation power (P_{exc}/P_{sat}). P_{sat} was calculated from the data shown in Figure SI8.*

As discussed earlier, to normalise optical excitation power we conduct PL intensity counts saturation and apply the power saturation model to extract P_{sat} value. Figure SI8 shows the obtained total counts per 532 nm excitation power, fit with the saturation curve that yields a P_{sat} value of 176 μW, which was earlier used for normalisation in Figure SI7e.

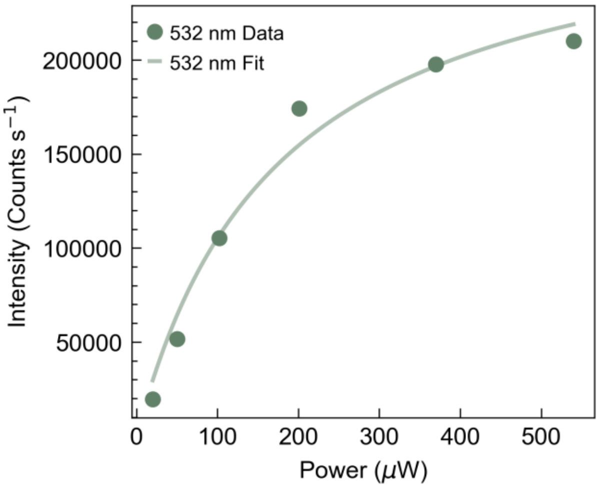

Figure SI8: Power saturation of the emitter with 703 nm ZPL. *PL intensities under varied optical power, with counts displaying saturation behaviour. Solid line corresponds to a fit using the model $I(P) = I_\infty P / (P + P_{sat})$. The extracted saturation power is 176 μW.*

Next, we examine another spin complex emitter with 718 nm ZPL. Its PL spectra and autocorrelation measurements shown in Figure SI9a-b for 532 nm and 633 nm excitations. Similar to the emitter with 703 nm ZPL discussed earlier in this section, this emitter also exhibits higher contrast under shorter wavelength excitation, with the CW-ODMR spectra in Figure SI9c showing a stronger signal for 532 nm compared to 633 nm excitation. As with the previously discussed emitter (703 nm ZPL), the power-dependent contrast under 633 nm excitation could not be reliably determined due to the poor signal-to-noise ratio. The high PL counts and robust ODMR contrast observed under 532 nm excitation allowed for the observation of the power-dependent contrast at this wavelength. For this emitter, the contrast appears to increase continuously with increasing laser power. This behaviour may be due to the much higher saturation power measured for this emitter, 798 μW, as determined from the optical power saturation curve shown in Figure SI10.

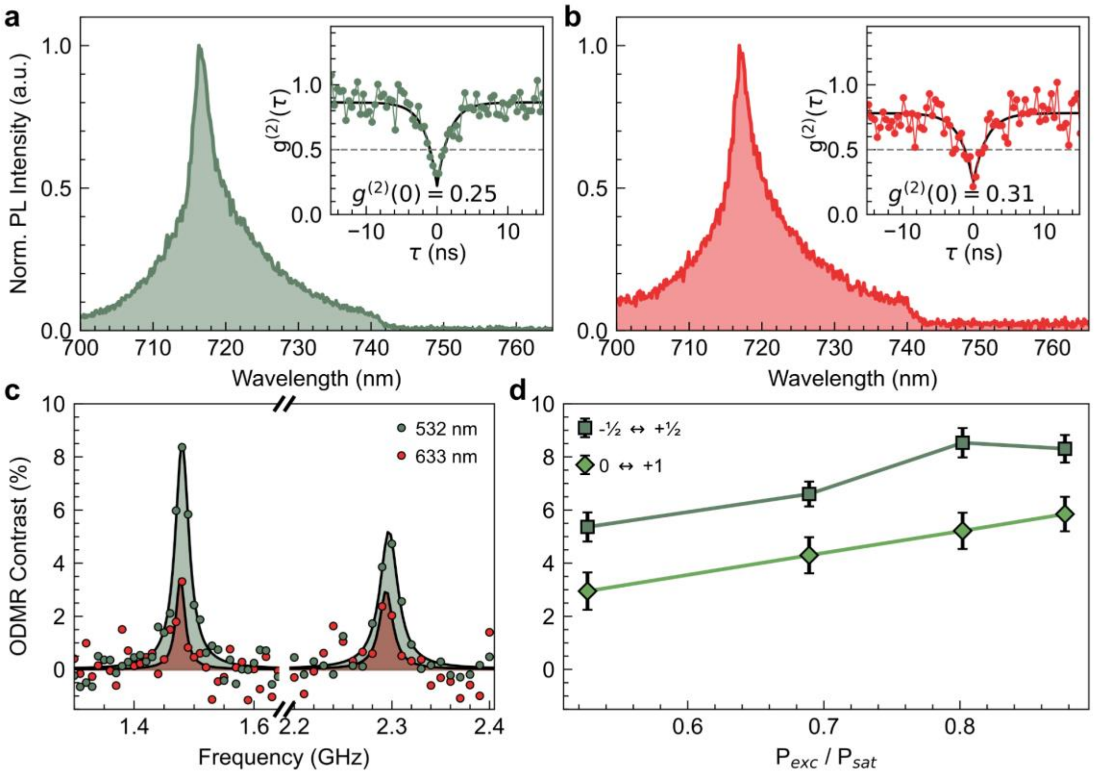

Figure SI9: PL and ODMR of emitter with 718 nm ZPL. (a) *PL spectrum of the emitter under* ***(a)*** *532 nm (green), and* ***(b)*** *633 nm (red) excitation. Inset: autocorrelation measurements under the different wavelength excitations.* ***(c)*** *CW-ODMR spectra of the emitter excited with 532 nm and 633 nm showing the relative contrast for the -½ ↔ +½, and 0 ↔ +1 transitions.* ***(d)*** *Measured ODMR contrast of the -½ ↔ +½ (squares) and 0 ↔ +1 (diamonds) transitions under 532 nm excitation plotted as a function of the excitation power normalised by the saturation power (P_{exc}/P_{sat}). P_{sat} was calculated from the data shown in Figure SI10.*

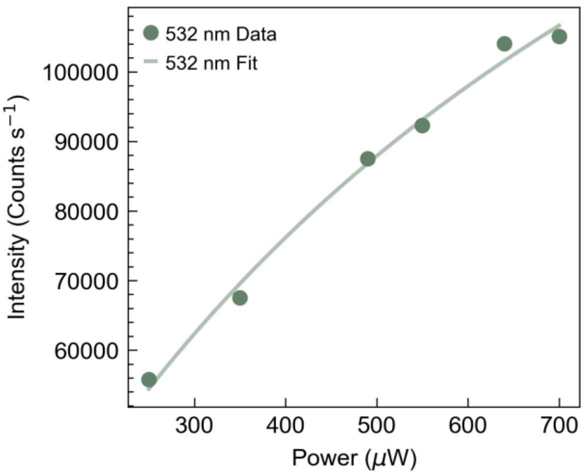

Figure SI10: Power saturation of the emitter with 718 nm ZPL. *PL intensities under varied optical power, with counts displaying saturation behaviour. Solid line corresponds to a fit using the model $I(P) = I_\infty P / (P + P_{sat})$. The extracted saturation power is 798 μW.*

Continuing examination of the hBN spin complex under varied excitation wavelengths, we study an emitter with ZPL centred at 672 nm. Its PL spectra and autocorrelation measurements under 488 nm and 532 nm excitation are shown in Figure SI11a-b. We note that this emitter could not be excited under 633 nm illumination. Both 488 nm and 532 nm excitation produce similar spectral features and autocorrelation behaviour. However, under 488 nm excitation no detectable CW-ODMR signal was observed. Similar to the previously discussed emitter, the CW-ODMR contrast increases continuously with increasing laser power. This behaviour is likely related to the high saturation power of this emitter (3.7 mW), as determined from the optical power saturation curve in Figure SI12. This emitter presents an intriguing possibility of optically exciting single defects while selectively suppressing spin-dependent behaviour depending on the excitation wavelength.

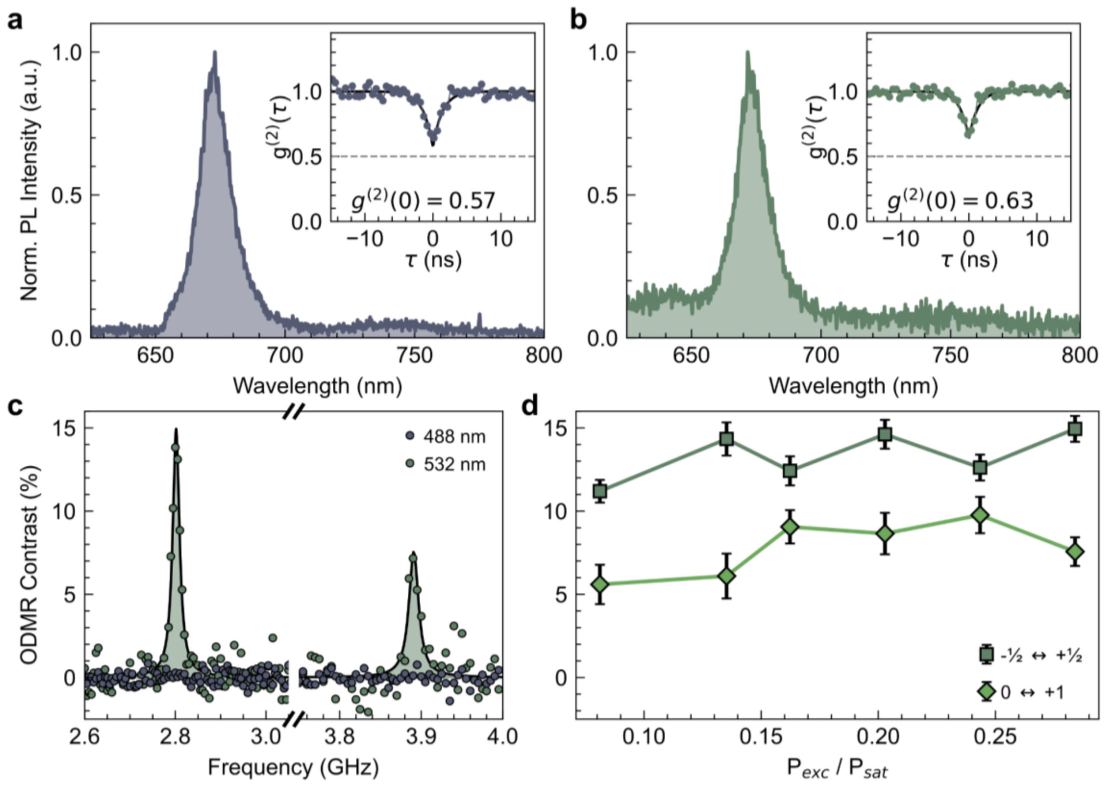

Figure SI11: PL and ODMR of the emitter with 672 nm ZPL. (a) *PL spectrum of the emitter under* ***(a)*** *488 nm (blue), and* ***(b)*** *532 nm (green) excitation. Inset: autocorrelation measurements under the different wavelength excitations.* ***(c)*** *CW-ODMR spectra of the emitter excited with 488 nm and 532 nm showing the relative contrast for the -½ ↔ +½, and 0 ↔ +1 transitions at ~100 mT. For the 488 nm excitation no ODMR was detected.* ***(d)*** *Measured ODMR contrast of the -½ ↔ +½ (squares) and 0 ↔ +1 (diamonds) transitions under 532 nm excitation plotted as a function of the excitation power normalised by the saturation power (P_{exc}/P_{sat}). P_{sat} was calculated from the data shown in Figure SI12.*

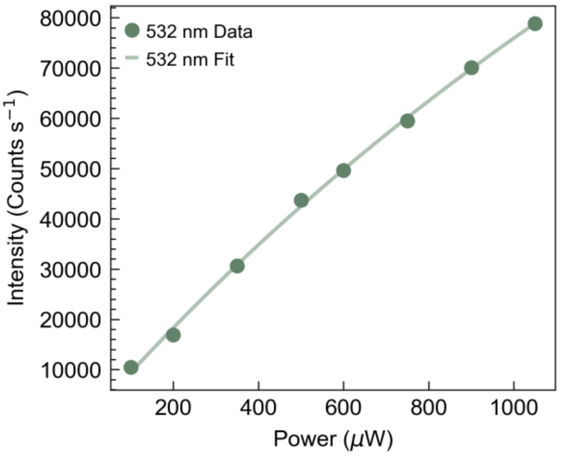

Figure SI12: Power saturation of the emitter with 672 nm ZPL. *PL intensities under varied optical power, with counts displaying saturation behaviour. Solid line corresponds to a fit using the model* $I(P) = I_\infty P / (P + P_{sat})$*. The extracted saturation power is 3.7 mW.*

To assess the generality of the observed excitation wavelength dependence, Table SI2 summarizes ODMR contrast values for multiple emitters with different ZPL positions and excitations. No clear correlation is observed and the studied spin complexes exhibit markedly different responses to excitation wavelength. However, all emitters within this 40 nm ZPL range consistently show that ODMR contrast is strongly dependent on the excitation wavelength, with variations of up to a factor of ~3×. This supports the proposed model of excited state dependent population dynamics.

Table SI2: Comparison of measured emitters.

Emitter	ZPL Wavelength (nm)	Excitation Wavelength : Max ODMR Contrast (Transition)
Main manuscript 295 K, Figure 2a	721	405 nm : No emission<br>532 nm : 36% (-½ ↔ +½)<br>633 nm : 98% (-½ ↔ +½)
Main manuscript 25 K, Figure 4a	721	532 nm : 23% (-½ ↔ +½)<br>610 nm : 86% (-½ ↔ +½)<br>650 nm : 70% (-½ ↔ +½)
Figure SI7	703	405 nm : No detected ODMR<br>532 nm : 9.6% (0 ↔ +1)<br>580 nm : 2.1% (0 ↔ +1)
Figure SI9	718	532 nm : 8.5% (-½ ↔ +½)<br>633 nm : 3.5% (-½ ↔ +½)
Figure SI11	672	488 nm : No detected ODMR<br>532 nm : 15% (-½ ↔ +½)

Section 4: Explanation of model fit to power dependent contrast.

To interpret the power-dependent ODMR contrast behaviour observed in the main text (Figure 2b) and in the supplementary materials (Figure SI4b, SI7e, SI9d, and SI11d), we employ a rate-equation model that involves population dynamics of the spin complex under varied optical excitation conditions (wavelength and power).

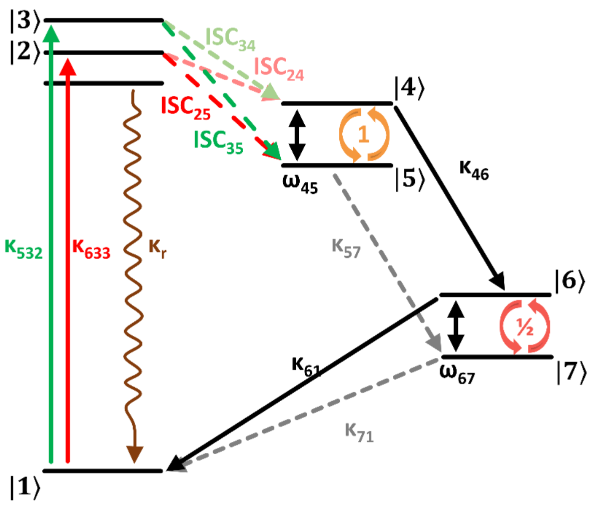

Figure SI13: The seven level rate model. *Simplified energy level diagram of the spin complex. The excitation rates are displayed as κ_{532} and κ_{633} for 532 and 633 nm excitation, respectively. The radiative decay rate (κ_r) is also shown in brown. The other transition rates are described as ISC_{ij}, κ_{ij} or ω_{ij}, where i and j are the initial and final electron levels of the transition, respectively. The two RF driven spin transitions for 0 ↔ +1 and -½ ↔ +½ are indicated with the circular arrows labeled 1 and ½, respectively.*

The rate model used in Figure 2b is based on the model introduced by Whitefield *et al.*[2], where two excited states (|2⟩ and |3⟩) were assumed to couple differently to the metastable state (Figure SI13). To capture this effect, total intersystem crossing (ISC) rates were set in a 5:1 ratio for |2⟩ and |3⟩, respectively. i.e. $ISC_{24}+ISC_{25}:ISC_{34}+ISC_{35}$. For both 532 and 633 nm excitation wavelengths, an initial increase in ODMR contrast is observed, followed by a reduction as the laser power increases. To reflect this phenomenon, power dependent ISC rates were introduced similarly to Dréau *et al.*[3] and in line with experimental observations by Patel *et al.*[4] As the laser power increases, the polarisation following ISC of the different triplet states converge. The ODMR contrast shown in Figure 2b was simulated with excitation rates (κ_{532} and κ_{633}) from $2x10^6$ to $4.6x10^8$. The power dependent ISC rates are defined by a linear relationship $ISC_{ij} = m\kappa_{532, 633} + b$, where m and b are for ISC_{ij} is shown in Table SI3.

Table SI3: Power dependent ISC rates as defined by the linear relationship $ISC_{ij} = m\kappa_{532, 633} + b$.

	ISC_{24}	ISC_{25}	ISC_{34}	ISC_{35}
m ($\times 10^{-4}$)	7.965	-7.965	1.593	-1.593
b	1600	630400	320	126079

This assumption appropriately predicts the rise and fall of contrast for both 532 and 633 nm excitation with increasing laser power.

The S = 1 triplet has been simplified to the $|4\rangle$ and $|5\rangle$ states for $|\pm1\rangle$ and $|0\rangle$, respectively. Similarly, $|6\rangle$ and $|7\rangle$ represent the states $|T_{\pm}\rangle$ and $|ST_0\rangle$ in the S = {1,0} singlet-triplet state. The transition rates between these two metastable states and the ground state are representative rates taken from the study in Whitefield *et al.*[2]. These rates are not assumed to be wavelength or laser power dependent and are shown in Table SI4.

Table SI4: Wavelength and laser power independent transition rates.

Laser Power Independent Transition Rates (s^{-1})						
κ_r	κ_{46}	κ_{57}	κ_{61}	κ_{71}	ω_{45}	ω_{67}
2×10^8	2×10^6	3×10^5	1×10^8	4×10^4	6×10^4	6×10^4

Section 5: Multi-wavelength co-excitation of emitter with 721 nm ZPL.

This section corresponds to Figure 3 of the main text that studied the spin complex using the co-excitation scheme. Since only two of the four spin complex transitions were covered in the manuscript, one from each of the spin manifolds (0 ↔ +1 of S = 1 and -½ ↔ +½ of S = {1,0}), here we show the behaviour of 0 ↔ -1 and -1 ↔ +1 (both transitions of S = 1) under the same co-excitation conditions used in the main text.

To begin, we confirm that the co-excitation setup reliably drives all four spin transitions using either one of two wavelength excitations. This is demonstrated as two ODMR spectra in Figure SI14a, where only a single excitation wavelength was used to optically drive the emitter with 721 nm ZPL. Note, amplitude of the 0 ↔ -1 transition appears lowest primarily due to the range limit of the microwave amplifier. Upon conducting the co-excitation, shown in Figure SI14b, we observe a similar trend as in the main text of this work. In particular, both 0 ↔ -1 and -1 ↔ +1 decrease in intensity with increasing 532 nm power, while keeping 633 nm power constant at 75 μW. Interestingly, the contrast change of the S = 1 transitions decreases at a slightly slower rate compared to the transition of the S = {1,0}. As example, in Figure SI14b the -1 ↔ +1 decreases to 16 % from 25 %, while in Figure 3g of the main text the -½ ↔ +½ falls from 53 % to 34 %. Studying this further in future works could unveil further details about operations of the spin complex emitters.

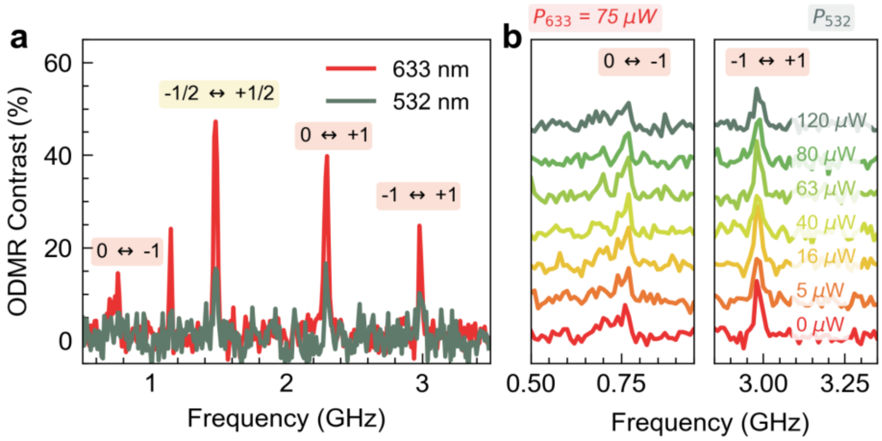

Figure SI14: Co-excitation ODMR of 0 ↔ -1 and -1 ↔ +1 transitions of emitter with 721 nm ZPL. (a) *Full ODMR spectra showing all spin complex transitions using co-excitation setup and only addressing the emitter with 200 μW of 532 nm or 75 μW of 633 nm.* ***(b)*** *Left and right panels correspond to 0 ↔ -1 and -1 ↔ +1 transitions, respectively, while 633 nm power was fixed at 75 μW and 532 nm power was varied from 0 to 120 μW.*

Section 6: Characterisation of emitter with 721 nm ZPL at 25 K.

This section extends the excitation-wavelength dependent ODMR analysis by examining the dependence of the spin-complex at cryogenic temperature (25 K). Performing these measurements at low temperature suppresses thermal broadening and background contributions, allowing a more direct comparison of ODMR contrast across different spin transitions and excitation wavelengths.

Since the main text already examines the -½ ↔ +½ and 0 ↔ +1 transitions, here we look at the -1 ↔ +1. Figure SI15a shows CW-ODMR spectra acquired under 532 nm, 610 nm, and 650 nm excitation. Similar behaviour is observed, where excitation wavelengths above 600 nm lead to a pronounced enhancement of ODMR contrast.

To directly compare the excitation-wavelength dependence for both spin manifolds, Figure SI15b summarises the extracted ODMR contrast values for the -½ ↔ +½, 0 ↔ +1, and -1 ↔ +1 transitions. All three transitions exhibit a consistent trend, with a sharp increase in contrast when moving from 532 nm to 610 nm excitation, followed by similarly high contrast at 650 nm. This indicates that excitation under longer-wavelengths promotes an electron to an energetic level with greater coupling to the metastable regime accessed via the ISC, thus resulting in higher populations of all spin transitions.

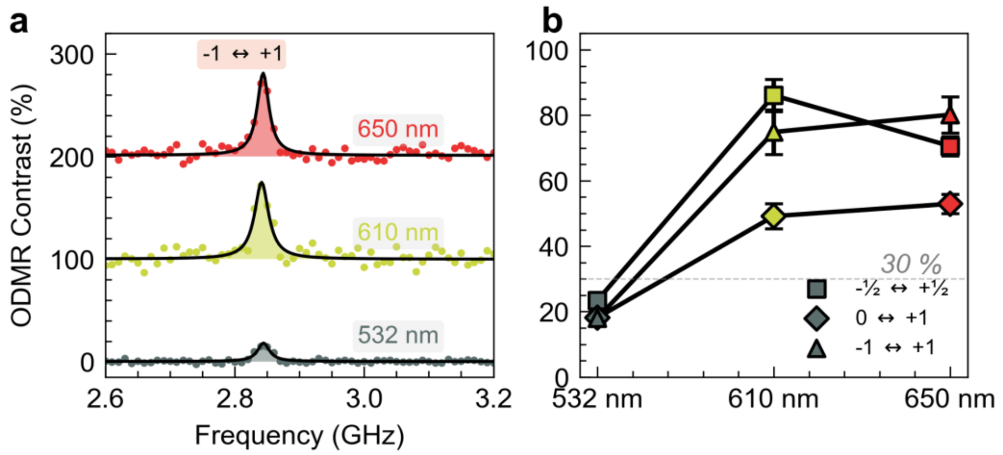

Figure SI15: Excitation-wavelength dependence of ODMR contrast at 25 K. (a) *CW-ODMR spectra of the −1↔+1 transition acquired under 532 nm, 610 nm, and 650 nm excitation, showing a strong enhancement of contrast for excitation wavelengths above 600 nm.* ***(b)*** *Summary of extracted ODMR contrast for the -½ ↔ +½, 0 ↔ +1, and -1 ↔ +1 transitions as a function of excitation wavelength, demonstrating an increased contrast at longer excitation wavelengths.*

To verify the spectral stability of the emitter at cryogenic temperature, time-resolved PL spectra were acquired at 25 K using consecutive 15 s integrations over a total duration of 300 s. A representative spectrum extracted from this dataset is shown in Figure SI15a, displaying two dominant emission features: a strong peak centred at 719 nm and a weaker at 724 nm.

The temporal evolution of the emission is shown in Figure SI16b as a spectra over time heatmap. Both spectral features remain stable over the full acquisition window, with no

observable spectral broadening, spectral diffusion, or intensity fluctuations. The emitter maintained stable optical characteristics at cryogenic temperature, suitable for subsequent PL ODMR measurements.

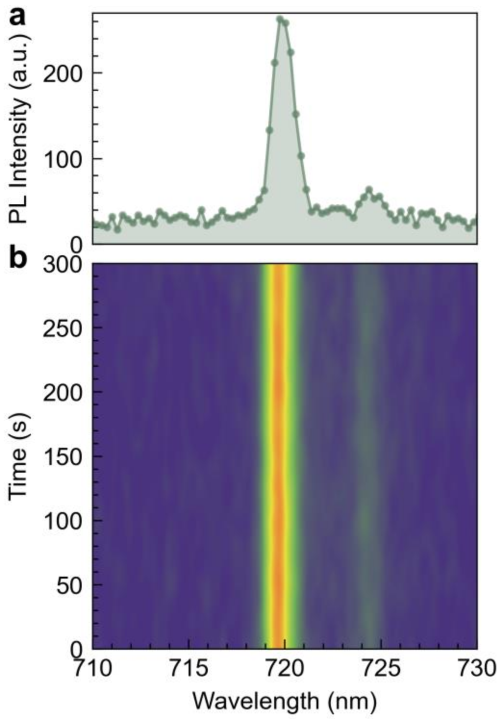

Figure SI16: Spectral stability of the emitter at 25 K. (a) *Representative PL spectrum of the emitter extracted from the time-resolved measurement, showing a dominant peak at 719 nm and a weaker peak at 724 nm.* ***(b)*** *Heatmap consists of consecutive PL spectra acquired with 15 s integration time over 300 s, demonstrating stable emission of both peaks.*

Section 7: PL ODMR of emitter with 721 nm ZPL at 295 K.

To examine whether the spectral response of the spin complex is preserved at room temperature, we performed PL ODMR measurements at ambient conditions (295 K) using the same analysis approach as employed at cryogenic temperature in the main text (Figure 4c,d). These measurements provide further insight into the influence of increased phonon coupling and thermal broadening on the spectral emissions.

Figure SI17a shows representative PL spectra acquired under identical optical excitation conditions with the microwave field set either on resonance with one of the -½ ↔ +½ or 0 ↔ +1 transitions, or detuned to an off resonant frequency. The two dominant emission features remain clearly resolvable and are fit with a triple Lorentzian model to account for additional phonon coupling at longer wavelengths. The extracted peak areas and their ratios are summarised in Figure SI17b. Similar to the cryogenic results, the area ratios between the two spectral components exhibit only minor variations between on- and off-resonant microwave excitation. The extracted ratios deviate by less than 0.06 across all measured conditions, indicating that both spectral components continue to couple evenly to the metastable regime at room temperature.

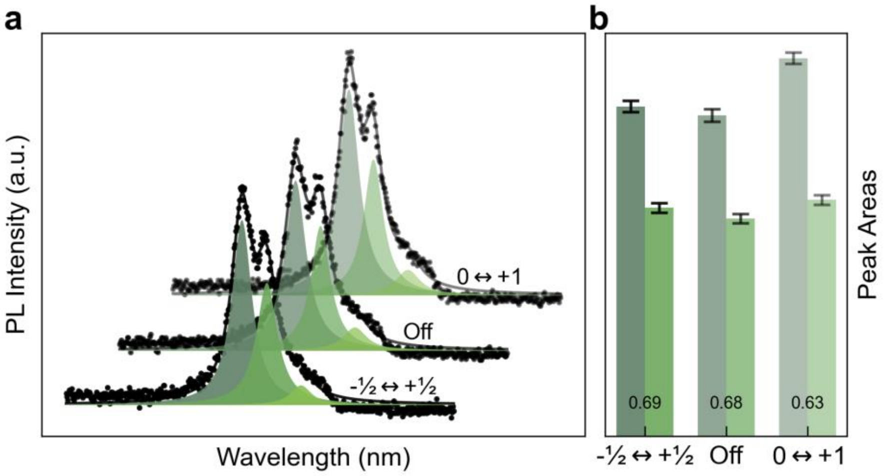

Figure SI17: PL-resolved ODMR of the 721 nm ZPL emitter at room temperature (295 K). ***(a)*** *Representative PL spectra acquired with the microwave field tuned on resonance with the -½ ↔ +½ or 0 ↔ +1 transitions, and off resonance for comparison. Shaded curves indicate triple Lorentzian fits used to extract peak areas.* ***(b)*** *Bar plots of extracted peak areas and corresponding ratios annotated for each microwave condition, showing minimal variation between on- and off-resonant excitation.*

The conducted analysis shows that PL ODMR remains a robust probe of spin dynamics even in the presence of stronger phonon interactions, and confirms that the spectral signatures observed at low temperature persist under ambient conditions.

Section 8: Direct Current Magnetic Field Sensitivity.

A quantum sensor operates via optical readout of its spin transitions whose degeneracy is lifted by an external field, such as magnetic. Changes in the resonance frequency induced through this Zeeman effect are measured by comparing PL acquired at each condition, thus allowing quantitative determination of applied fields. In CW-ODMR, the recorded spectrum can be expressed as a resonance dip characterised by its contrast C, linewidth $\Delta\nu$, and photon detection rate R. Assuming shot-noise limited photon detection, the direct current (DC) magnetic field sensitivity is defined as[3]:

$$\eta_{DC} = \mathcal{P}_L \frac{h}{g_e\mu_B}\frac{\Delta\nu}{C\sqrt{R}}$$

where h is Planck's constant, g_e is the electron g-factor, μ_B is the Bohr magneton, and P_L is a numerical factor determined by the lineshape (e.g., $P_L \approx 0.77$ for a Lorentzian profile) . This expression highlights that optimal sensitivity requires a narrow linewidth, high ODMR contrast, and large photon count rate.

Extending on the analysis presented in the main text of this work, to further quantify the sensing performance of the spin complex, here we analyse the dependence of ODMR linewidth ($\Delta\nu$) on optical excitation power. Following from the above formulation, the DC magnetic field sensitivity scales linearly with linewidth ($\eta_{DC} \propto \nu$), thus a plot of its power dependence is essential for evaluating optimal operating conditions.

Figure SI18 shows the extracted linewidths for the -½ ↔ +½ and 0 ↔ +1 transitions as a function of normalised optical excitation power using conditions defined in Section 2 of this document. Under 532 nm excitation (Figure SI18a), the linewidth remains stable across the explored power range, with only minor variations within the uncertainty. In contrast, Figure SI18b shows 633 nm excitation which exhibits power dependence, starting with broader linewidths at low excitation and progressively narrowing with increased power and significantly below the values of 532 nm excitation.

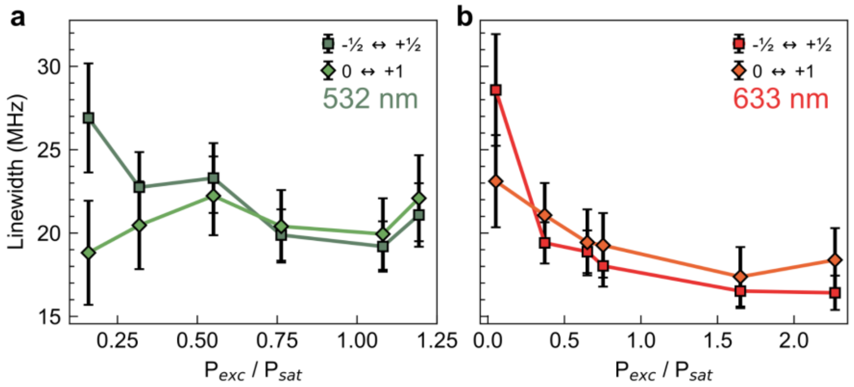

***Figure SI18: ODMR linewidth dependence on excitation power.** ODMR linewidths extracted from Lorentzian fits for the -½ ↔ +½ and 0 ↔ +1 transitions as a function of normalised excitation power under (a) 532 nm and (b) 633 nm excitations.*

This behaviour directly impacts the achievable sensitivity. Using the measured contrast, linewidth, and photon count rates. The calculated DC magnetic field sensitivity as a function

of excitation power for both wavelengths is shown in Figure SI19. For 532 nm excitation, the relatively constant linewidth combined with moderate contrast results in a gradual improvement in sensitivity with increasing power, largely attributed to increased photon counts at the detector. On the other hand, 633 nm excitation benefits from both higher ODMR contrast and reduced linewidth within the whole examined power range, leading to a significantly improved sensitivity for both transitions (compared to the 532 nm datasets).

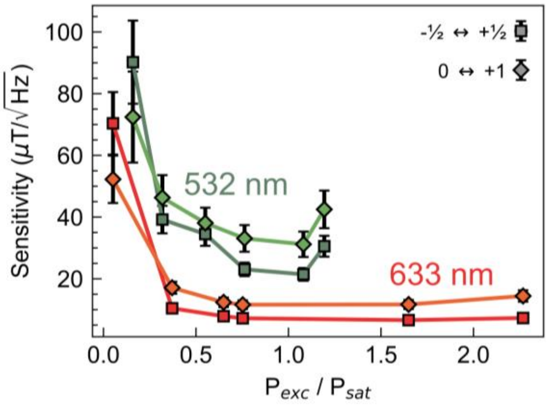

Figure SI19: DC magnetic sensitivity as a function of excitation power. *Calculated DC magnetic sensitivity using measured contrast, linewidth, and photon count rate for both excitation wavelengths at different powers.*

These results highlight that excitation wavelength plays a critical role not only in determining ODMR contrast but also in governing linewidth broadening mechanisms. The combination of enhanced contrast and reduced linewidth under 633 nm excitation results in superior magnetic field sensitivity compared to 532 nm excitation.

Although 633 nm excitation yields significantly improved sensing performance, it also introduces pronounced blinking, as discussed in the main text. Since the DC magnetic field sensitivity assumes a continuous photon detection rate, intermittency reduces the effective measurement time. In practice, this is characterised as a reduced duty cycle of a quantum sensor, since the achievable sensitivity scales with the effective photon rate rather than the time averaged emission. Thus, blinking directly affects sensitivity through the dependence $\eta_{DC} \propto 1/\sqrt{R}$.

To account for this effect, we extract the effective sensing duty cycle directly from the PL time traces (Figure 3 of the main text). For each co-excitation condition, a threshold count level I_{th} is defined from the counts histogram, where the $I_{th,0}$ minima was defined to separate the signal into a dark state ($I < I_{th}$) and a bright state ($I \geq I_{th}$). The bright-state fraction is then obtained as $f_B = N_B / N = 1 - f_D$, where N_B is the number of time bins that are above threshold, N is the total number of bins, and f_D is the corresponding dark state fraction. Following, bright state photon count rate is calculated as the conditional mean of the counts above threshold,

$$R_B = \frac{1}{N_B} \sum_{i \in B} i$$

Where I_i is the measured photon count rate in the i-th time bin and the sum runs only over bins assigned to the bright state. The effective count rate that is only relevant for sensing is

therefore $R_{Eff} = f_B R_B$, which accounts for both the intrinsic brightness of the emitter and the fraction of time used for sensing. This yields an updated expression where sensitivity scales with effective count rate as $\eta_{DC} \propto 1/\sqrt{(R_B f_B)}$. Using this formulation, in Figure SI20 we evaluate the 633 nm and 532 nm co-excitation scheme on the sensing performance of the emitter with 721 nm ZPL.

Comparing the mean count rate ($R\sigma$) and effective count rate (R_{Eff}) in Figure SI20a, we observe that R_{Eff} is slightly reduced relative to the average count rate at lower 532 nm powers (<50 μW). This behaviour is consistent with the presence of blinking, where dark state occupation reduces the usable photon rate. As the 532 nm power increases, blinking is progressively suppressed, leading to a convergence between R_{Eff} and R_σ.

Correspondingly, Figures SI20b-d show that the impact on sensitivity is minimal at low 532 nm excitation powers (<40 μW). For example, at 0 μW, 5 μW, and 16 μW, using the averaged count rate yields slightly lower sensitivity values. However, when corrected using the effective count rate, the sensitivity increases by 0.26 $\mu T\sqrt{Hz}^{-1}$, 0.11 $\mu T\sqrt{Hz}^{-1}$, and 0.10 $\mu T\sqrt{Hz}^{-1}$ for -½ ↔ +½ transition, and by 0.41 $\mu T\sqrt{Hz}^{-1}$, 0.16 $\mu T\sqrt{Hz}^{-1}$, and 0.13 $\mu T\sqrt{Hz}^{-1}$ for 0 ↔ +1 transition. These results suggest that although blinking reduces the effective sensing duty cycle and is non-negligible, its effect remains minimal.

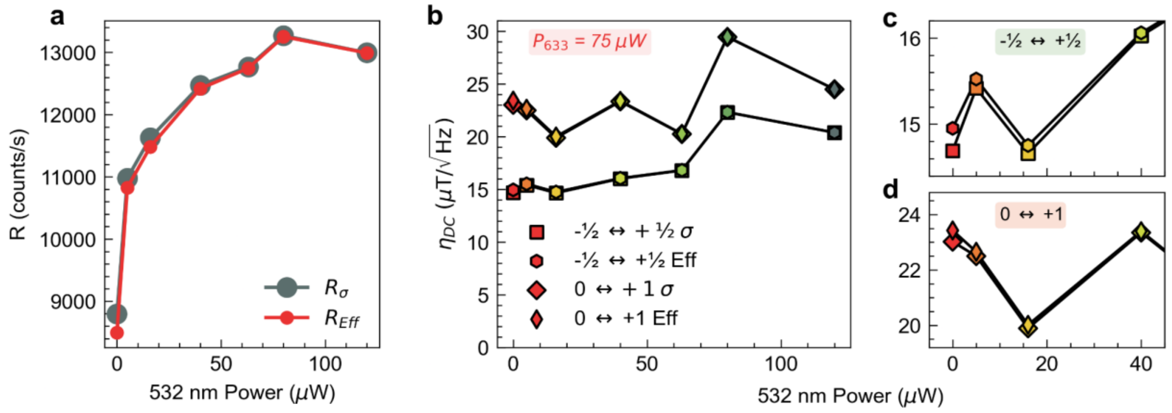

Figure SI20: Influence of blinking on DC magnetic field sensitivity. (a) *Comparison between mean photon count rate (R_σ) and effective count rate (R_{Eff}) as a function of 532 nm excitation power. (b) Extracted DC magnetic field sensitivity calculated using both, R_σ and R_{Eff} for the -½ ↔ +½ and 0 ↔ +1 transitions. (c,d) Sensitivity plots for the same transitions, but zoomed into a low power regime of 532 nm excitation. For all measurements 633 nm power was kept constant at 75 μW.*

Notably, the lowest sensitivity values are achieved at low co-excitation powers around 16 μW. This indicates that using 633 nm as main excitation to maximise ODMR contrast, combined with a small amount of 532 nm to suppress blinking, provides the most optimal balance between contrast enhancement and sensing duty cycle. The co-excitation scheme therefore yields the most favourable sensing performance, maximising effective photon collection while maintaining high spin readout contrast.

Section 9. Ground State Depletion Microscopy:

The spatial resolution of standard confocal microscopy used to address single quantum emitters is fundamentally limited by light diffraction and characterised by the relation $\lambda/(2n\,sin\alpha)$, where λ is the excitation wavelength and $n\,sin\alpha$ is the numerical aperture (NA) of the imaging objective. For a confocal system with 0.9 NA this corresponds to a resolution of ~300 nm at 532 nm excitation and ~350 nm at 633 nm excitation. As quantum emitters are point sources at the atomic scale, confocal microscopy does not provide true single atom spatial resolution for quantum sensing.

Recent developments in super resolution microscopy overcome this limitation by exploiting internal energy structure of emitters. One such approach, ground state depletion (GSD) microscopy, employs a Gaussian excitation beam together with a doughnut shaped depletion beam. For emitters with a long lived metastable state, this allows optical transfer of population into a dark state everywhere except at the intensity minimum of the doughnut beam. With this technique, the achievable lateral resolution scales with the depletion saturation intensity, resulting in resolution expressed as,[5]

$$\Delta r \approx \frac{\lambda}{2NA}\frac{1}{\sqrt{1+I/I_s}}$$

where I is the depletion intensity at the doughnut crest and I_s is the saturation intensity at which half of the emission is depleted. The I_s is governed by the efficiency of shelving into the metastable dark state, and scales inversely with both the excited fluorescent state lifetime and the effective cross section. The resolution dependence can then equivalently be expressed in terms of optical power,[6]

$$\Delta r \propto \frac{1}{\sqrt{1+P/P_s}}$$

such that P is the applied optical power and P_s is the depletion saturation power at which emitter brightness is reduced by half. In the absence of a dedicated GSD measurement, we approximate the relative depletion efficiency using the experimentally measured excitation saturation powers P_{sat}. These are not identical to P_s but rather provide an indication of a system's efficiency to be driven into a saturated or depleted regime.

In this work, the emitter with 721 nm ZPL showed saturation powers reduced from $P_{sat,532} = 151\ \mu$W to $P_{sat,633} = 97\ \mu W$. Thus, the expected resolution improvement can be estimated as

$$\frac{\Delta r_{633}}{\Delta r_{532}} \approx \sqrt{\frac{P_{sat,633}}{P_{sat,532}}} \approx \sqrt{\frac{97}{151}} \approx 0.80$$

which results in a 1.25 improvement. Given that typical GSD resolution in this wavelength range for hBN emitters is 50-100 nm[7], reduction in saturation power from 532 nm excitation with 80 nm resolution yields $\Delta r_{633} \approx 0.8 \times \Delta r_{532} \approx 60\text{-}65$ nm, which could be reduced further via the co-excitation scheme. This analysis proves GSD microscopy as a powerful tool for advancing towards nanometre scale quantum sensing.

References

1. Robertson, I. O. et al. A charge transfer mechanism for optically addressable solid-state spin pairs. Nature Physics 21, 1981-1987, doi: 10.1038/s41567-025-03091-5 (2025).

2. Whitefield, B. et al. Photodynamics and temperature dependence of single spin defects in hexagonal boron nitride. ACS Nano 20, 6200-6207 (2026).

3. Dréau, A. et al. Avoiding power broadening in optically detected magnetic resonance of single NV defects for enhanced dc magnetic field sensitivity. Physical Review B 84, 195204 (2011).

4. Patel, R. N. et al. Room temperature dynamics of an optically addressable single spin in hexagonal boron nitride. *Nano Letters* **24**, 7623-7628 (2024).

5. Bretschneider, S., Eggeling, C. & Hell, S. W. Breaking the Diffraction Barrier in Fluorescence Microscopy by Optical Shelving. *Physical Review Letters* **98**, 218103 (2007).

6. Han, K. Y., Kim, S. K., Eggeling, C. & Hell, S. W. Metastable Dark States Enable Ground State Depletion Microscopy of Nitrogen Vacancy Centers in Diamond with Diffraction-Unlimited Resolution. *Nano Letters* **10**, 3199–3203 (2010).

7. Kianinia, M. et al. All-optical control and super-resolution imaging of quantum emitters in layered materials. Nature Communications 9, 874 (2018).
